# Explore the correlations of occupational commitment, psychological resilience, job satisfaction, and burnout in kindergarten teachers’ turnover intentions in rural China

**DOI:** 10.3389/fpsyg.2025.1605831

**Published:** 2025-06-27

**Authors:** Yanan Zheng, Xiaoyu Wang, Li Yang

**Affiliations:** College of Education Science, Harbin Normal University, Harbin, China

**Keywords:** kindergarten teachers, turnover intention(s), occupational commitment, psychological resilience, job satisfaction, burnout

## Abstract

Ensuring the stability of teachers is a crucial task in developing early care education; thus, reducing the turnover rate of kindergarten teachers is the core priority. This research explores the correlation of four antecedents with teachers’ turnover intentions: occupational commitment, psychological resilience, job satisfaction, and burnout. This study utilized a stratified sampling methodology based on regional GDP levels and implemented a hybrid data collection approach combining online and offline questionnaire distribution to ensure comprehensive representation across socioeconomic contexts. Taking the collected data from 920 kindergarten teachers in the rural area of Hebei Province, China as the research object, the research presents the following findings: (i) nearly 60% of kindergarten teachers prefer to Move rather than Leave when having turnover intentions; (ii) the improvement of psychological resilience and working conditions could effectively mitigate the decision of kindergarten teachers to leave entirely; (iii) teachers with no turnover intentions are more concerned about intrinsic motivation, but there is a risk of “false retention”; (iv) job satisfaction has the most substantial impact on kindergarten teachers’ turnover intentions, but the performance is relatively poor; (v) risk and protective factors synergistically affect teachers’ turnover intentions.

## Introduction

The impact of teacher turnover on education can be devastating (Arthur and Bradley, 2023; Zhao et al., 2022). Especially concerning education, the teacher turnover rate is higher than in any other sector, which is an extremely serious occupational hazard (Räsänen et al., 2020). The exploration and analysis of turnover has become increasingly important in the teaching profession, which is common around the globe and extensive research has already been done (He et al., 2023; Caudal, 2022; Schaack et al., 2022; See et al., 2020). High turnover rates in the education field is a globe problem and occurs worldwide (Gundlach et al., 2024). For example, in the Louisiana, United States, approximately 40% of early childhood teachers quit their jobs every year and nearly one-third of centers experienced high turnover (Doromal et al., 2022). In Australia, the annual turnover rate of some childcare institutions reaches as high as 60% (Jovanovic, 2013). The same situation also occurs in China. In 2020, the turnover rate of teachers in private kindergartens in China reached 41% (Yang et al., 2021). High turnover rates of preschool teachers in rural China are more obvious (Guo and Li, 2023; Li and Gong, 2020). What’s more, a survey conducted across 12 provinces in China showed that almost 83.1% of kindergartens failed to meet the minimum ratio of preschool teachers to children (1:7) in rural areas (Yu et al., 2017) and this ratio has reached 1:17 in 2021 (Guan, 2021). Moreover, the attrition of preschool teachers in rural regions has become a common social phenomenon trapped in an unretained dilemma (Wang et al., 2022). Worse, the shortage of kindergarten teachers in rural China reached 640,000 (Liu and Bai, 2021). High turnover rates of kindergarten teachers could threaten the quality and stability of teaching staff, impair children’s early development, restrict the development of preschool education (Wells, 2015; Schaack et al., 2022), and lead to poorer student outcomes and achievement (Ronfeldt et al., 2013; Buchanan et al., 2013). Additionally, Hu et al. (2016) also point out that kindergarten teachers in rural China could not get enough and deserved attention and support, which has dramatically lagged far behind that of urban institutions. Therefore, it is urgent to investigate kindergarten teachers’ turnover and corresponding determinants and antecedents (Ren et al., 2024).

Turnover intention, referring to the inclination to leave the profession or quit the job (Tett and Meyer, 1993), has been regarded as an effective predictor of actual turnover behavior (Nguyen et al., 2022; Callado et al., 2023) and indicates a tendency toward turnover (Park et al., 2024). Continuous pressures and demanding job tasks increase turnover intentions (Wazir and Jan, 2020). Teachers’ turnover intention is regarded as the intention to leave the teaching profession (Matthews et al., 2022). Previous studies have investigated various antecedents which affect teachers’ turnover intentions (Collie, 2025; Gundlach et al., 2024; Liu, 2024; Van Eycken et al., 2024; Collie, 2023; Esop and Timms, 2019; Conley and You, 2009; Grant et al., 2019). Reviewing existing studies on teachers’ turnover intentions, we found that various antecedents were explored with turnover intentions in pairs. Moreover, there have been only a few empirical studies to investigate kindergarten teachers’ turnover intentions in rural China. Additionally, research on kindergarten teachers’ turnover has yet to distinguish actual outcomes of turnover intentions of moving to other kindergartens but still in early care education and leaving the field entirely. This study explores and examines occupational commitment, psychological resilience, burnout, and job satisfaction as predictors of kindergarten teachers’ turnover intentions in rural China.

### Occupational commitment

Commitment refers to the attachment between workers and their careers and the relationship to a particular organization (Meyer et al., 1993). It should be noted that regarding commitment literature, several terms, including occupation (Young et al., 2023; Collie et al., 2020; Lin et al., 2021), profession (Ilyas et al., 2023), and career (Chen and Zhang, 2023; Son and Kim, 2021), have been used somewhat interchangeably. This study would like to explore and assess commitment to a particular work field—early care education (ECE). Therefore, the term occupation is more appropriate, and occupational commitment will be discussed in this research. Lee et al. (2000) defined occupational commitment as the psychological link between an individual and their occupation based on an affective reaction to that occupation. Thus, we can imagine that workers or employees with higher occupational commitment have positive and affective feelings and active pursuit for their professional development (Liu et al., 2021), which could be regarded as a crucial motivation for individuals’ positive organizational behavior (Bakker and Schaufeli, 2008) and superb work performance (Meyer et al., 1989). Prior studies have suggested that occupational commitment is not only associated with a variety of positive outcomes, including low rates of absenteeism and higher work engagement (Freund, 2005) but is also inversely linked with intentions to leave a profession (Antony et al., 2024; Hackett et al., 2001). Additionally, the study of Hackett et al. (2001) also demonstrated that intention to leave the occupation is empirically distinguishable from the level of occupational commitment; theoretically, the two share a causal relationship, with commitment preceding quitting intention. Moreover, occupational commitment is also reported to be a crucial factor in decreasing actual turnover in existing studies (Antony et al., 2024; Callado et al., 2023; Lee et al., 2000; Meyer et al., 2002).

### Psychological resilience

Prior studies and research literature reflect little consensus about definitions of resilience, with substantial variations in operationalization and measurement of critical constructs (Luthar et al., 2000; Bonanno et al., 2015; Kalisch et al., 2015). Psychological resilience may be viewed as a measure of stress-coping ability and, as such, could be an essential target of treatment in anxiety, depression, and stress reactions (Connor and Davidson, 2003), which represents one such variable that has been found to reduce feelings of burnout (Johnson and Brown, 2013; Richards et al., 2016). It is measured in terms of both emotion regulation and coping strategies (Compas et al., 2017; Gross, 2015; Lazarus, 2000). Some scholars argue that psychological resilience is domain-specific, which may manifest differently in different domains (Todt et al., 2018). Therefore, it is vital to understand how psychological resilience manifests in teachers. Fortunately, understanding teachers’ life and career decisions from the perspective of psychological resilience has attracted wide attention (Lee and Sung, 2023; Zhi and Derakhshan, 2024; Chen, 2024; Brunetti, 2006; Castro et al., 2010; Gu and Day, 2007; Howard and Johnson, 2004; Kirk and Wall, 2010; Oswald et al., 2003). Additionally, more scholars and researchers are paying attention to teachers’ psychological resilience to understand teachers’ job satisfaction (Wang et al., 2024; Han, 2022; Brunetti, 2006; Kitching et al., 2009), teacher burnout and stress (Begum et al., 2024; Colomeischi and Ursu, 2024; Liu et al., 2021; Howard and Johnson, 2004), and career decision-making (Lee and Sung, 2023; Gu and Day, 2007). Several studies also suggested that psychological resilience may be the key to understanding teachers’ turnover, given identical and often dissatisfying and stressful working conditions (Liu et al., 2021; Beltman et al., 2011) For teachers, psychological resilience could explain coping with the less satisfying aspects of their work-life to become more committed to teaching over the long term (Beltman et al., 2011; Hong, 2012; Mansfield et al., 2012; Tait, 2008).

### Job satisfaction

Job satisfaction, simply put, refers to what people think about their jobs (Spector, 2022) or an attitude toward the job resulting from a cognitive evaluation of specific job aspects (Spector, 2022; Weiss, 2002). Across a variety of occupations, job satisfaction has been found to have an impact on a variety of outcomes for it is not only crucial for employees’ wellbeing and productivity but also plays as a pivotal role in the health of the employer-employer relationship (Jogi et al., 2025; Redondo et al., 2021; Hsiao et al., 2020). The link between job satisfaction and turnover gets the most attention. Many researchers have theorized that job satisfaction is a crucial antecedent of employee turnover (Jogi et al., 2025; Mobley et al., 1979; Williams and Hazer, 1986), and job satisfaction is found to be negatively associated with turnover intention (Chiho, 2022; Redondo et al., 2021; Hsiao et al., 2020; Hom and Kinicki, 2001; Lee et al., 1996; Michaels and Spector, 1982; Mobley et al., 1979). That is, when employees are dissatisfied with their work, they will have lower work motivation, which will decrease the organization’s overall performance. Moreover, higher job satisfaction can positively sustain great work productivity (Akosile and Ekemen, 2022) and push them to make new accomplishments (Mardanov, 2021). Conversely, a lack of satisfaction can lead to opposite outcomes, such as work stress and burnout. Additionally, Ostroff (1992) found that organizations with more satisfied employees tend to be more effective than those with dissatisfied employees. In the area of education, previous studies have found a negative correlation between teacher turnover and job satisfaction (Shah and Jumani, 2015). For teachers, job satisfaction predicts retention and performance of teachers (Chen et al., 2020) and higher job satisfaction could reduce the turnover (Ren et al., 2024). In this regard, it is vital to consider the role of job satisfaction in the teaching profession (Wartenberg et al., 2023), which could link to the different outcomes such as turnover intentions ((Li and Yao, 2022; Madigan and Kim, 2021).

### Burnout

Burnout is a set of symptoms or physical conditional of emotional exhaustion, cynicism, depersonalization, and personal accomplishment that occurs frequently among individuals when they feel they can no longer give of themselves at a psychological level in response to working stress (Santi et al., 2020; Maslach and Jackson, 1981). Burnout is categorized into three dimensions: emotional exhaustion, depersonalization, and reduced personal accomplishment (Maslach and Jackson, 1981; Schaufeli et al., 2009). Burnout can mean unnecessary and demanding tasks for employees (Wazir and Jan, 2020). Maslach and Leiter (2016) have pointed out that burnout has been recognized as an occupational hazard in various human-centered occupations, such as human services, education, and health care. According to prior research, job burnout has a strong positive relationship with turnover intention (Srivastava and Agrawal, 2020) whereas it has a negative relationship with job satisfaction (Skaalvik and Skaalvik, 2020; Wong and Spence Laschinger, 2015; Lu and Gursoy, 2013) and occupational commitment (Kresna and Putra, 2020). In the context of education, teaching is an emotionally draining and stressful profession (Kyriacou, 2001), and burnout appears to be a relatively common experience in education occupations (Parmar et al., 2022). Most studies and researchers also regard burnout as a state of emotional, physical, and attitudinal exhaustion that may develop in teachers who have been unsuccessful in coping effectively with stress over a long period (Guglielmi and Tatrow, 1998), which will lead to different outcomes: decreased job satisfaction (Skaalvik and Skaalvik, 2010), reduced teaching self-efficacy (Roohani and Iravani, 2020) and an increased decision or desire to leave the job or profession and reduced job performance (Parmar et al., 2022; Perrone et al., 2019). Burnout of kindergarten teachers would inevitably increase the kindergarten teacher turnover (Chen et al., 2023). Organizations should make strategies and policies to reduce burnout and to prevent burnout to be able to perform better (Tanculescu-Popa, 2020).

### Hypotheses of this study

In summary, the existing research provides a preliminary understanding of the relationship of turnover intentions with four factors, respectively. In the current study, we investigated the relationship between occupational commitment, psychological resilience, job satisfaction, burnout, and teachers’ turnover intentions to figure out the validity and reliability of these four factors together and turnover intentions among kindergarten teachers in rural China. Based on the current analysis, several hypotheses are made:

H1: Occupational commitment in rural kindergarten teachers is negatively related to their turnover intentions.

H2: Rural kindergarten teachers’ psychological resilience negatively predicts their turnover intentions.

H3: Burnout of rural kindergarten teachers has a positive relationship with their turnover intentions while a negative relation with job satisfaction.

H4: Occupational commitment and job satisfaction of rural kindergarten teachers have an inverse effect on their turnover intentions.

## Materials and methods

### Participants

The survey procedures begin with a stratified sampling framework based on regional GDP levels. First, counties are categorized into high-, medium-, and low-GDP tiers using official economic data, ensuring systematic representation of diverse socioeconomic contexts. Within each tier, representative counties are randomly selected to mitigate geographic bias. Subsequently, a multi-stage sampling approach is implemented: rural kindergartens are randomly chosen from these counties, followed by the recruitment of teachers within each kindergarten, with attention to demographic diversity in experience, position, and educational background. To accommodate regional disparities in infrastructure, a hybrid data collection strategy (online and offline mix) is employed in this study. This study strictly adhered to academic research ethics guidelines. All participants were fully informed in writing and electronically about the research purpose, procedures, and data usage, and approval was obtained from the university’s ethics committee. Finally, we selected kindergarten teachers in the rural area of Hebei Province, China, as the research object, and these teachers were from 54 public kindergartens, 5 public-private kindergartens, and 20 private kindergartens. The total number of distributed questionnaires was 1,020, and 956 questionnaires were recovered. However, 36 participants were excluded after the data collection because of (a) a regular pattern of responses; (b) significant missing data; and/or (3) contradictory responses to relevant items. Thus, 920 valid questionnaires were included in the subsequent analyses.

The demographic descriptive statistics of the sample are shown in Table 1. Within our sample of teachers, 58% were married, 71.1% were from public kindergartens, 46.1% of teachers had no establishment, and the average income of the sample was about RMB 1,600 yuan. Regarding the education variable, teachers with technical secondary degrees accounted for 45.6%. The average number of years working in the early care education industry was more than 7 years, and the average length of service in current kindergartens was about 4 years. The demographic descriptive statistics of the sample were generally consistent with the findings from the Educational Statistics Yearbook of China (2022), except our sample had a higher education background than the national average level. The demographic descriptive statistics show that the surveyed sample was reasonable with a strong explanatory power.

**TABLE 1:** Demographic descriptive statistics of kindergarten teachers.

Variable	Items	*N*	%	Variable	Item	*N*	%	Variable	Item	*N*	%
Marital status	Married	533	58	Type of kindergartens	Public	653	71.1	Income (RMB)	<1,000	128	13.9
	Unmarried	387	42		Public-private	20	2.2		1,000–2,000	292	31.7
Education	Technical secondary degree	419	45.6	Establishment	Private	246	26.7	Position	2,000–3,000	327	35.6
	Associate degree	485	52.8		Yes	496	53.9		>3,000	173	18.9
	Bachelor’s degree or above	16	1.7		No	424	46.1		Headteacher	526	57.2
Length of service (current kindergarten)	0–5	522	56.7	Years of working (early care education industry)	0–5	450	48.9		Assistant teacher	282	30.6
	6–10	132	14.4		6–10	148	16.1		Leader	112	12.2
	11–15	113	12.2		11–15	168	18.3				
	16–20	66	7.2		16–20	72	7.8				
	21 +	87	9.4		21 +	82	8.9				

Establish has the same meaning as bianzhi, the bureaucratic benefits for government workers.

### Methodology

Given the relevant research conducted by scholars in China and abroad, we determined the research framework and developed the research tools. At the level of job requirements, we chose several indicators such as organizational management, labor reward, work itself, emotional regulation, and coping ability. The level of job resources included several indicators, such as remuneration and incentives, self-development, policy support, and emotional status. Finally, this study determined 29 first-level indicators and 53 determinants, and the principal components were extracted by principal component analysis. According to the steep slope diagram and the structure of determinants after turning the axes (see Table 2), this study finally determined four common factors that were consistent with the constructs and items of the survey.

**TABLE 2:** Mean value of the predictors.

Variables (*n* = 243)	Mean	SD	Variables (*n* = 243)	Mean	SD
Occupational commitment	3.68	0.71	Burnout	3.06	0.57
Normative commitments	4.36	0.80	Emotional exhaustion	3.12	1.05
Emotional commitment	3.63	0.91	De-personalization	2.01	0.95
Ongoing commitment	3.04	0.92	Self-efficacy	4.04	0.78
Psychological resilience	3.80	0.54	Job satisfaction	3.61	0.64
Cognitive reassessment	4.08	0.68	Satisfaction with the job itself	3.78	0.84
Expression inhibition	3.13	0.95	Internal job satisfaction	3.98	0.73
Programs	4.07	0.72	Return satisfaction	3.25	0.89
Responding to	4.42	0.64	Organizational management satisfaction	3.66	0.99
Tool support	3.49	0.88	Satisfaction with the practice environment	3.40	0.46
Coping strategies	3.99	0.56			
Emotional regulation	3.60	0.66			

Based on the mentioned above, the questionnaire included three parts. The first part was demographic variables, mainly including teachers’ marital status, length of service in the current kindergarten, years working in the early care education industry, establishment, type of kindergartens, income, and education. The second part was the questionnaire on teachers’ turnover intentions. The third part was the determinants scale, which consisted of the occupational commitment scale, psychological resilience scale, job satisfaction scale, and burnout scale. The total number of items was 62, and the details of the questionnaire and each scale were as follows:

1: Questionnaire on Teachers’ Turnover Intentions

This questionnaire aimed to determine the teachers’ turnover intentions; thus, we designed the following question.

“Which is more in line with you?”

A. I want to stay in the same jobs (*you are a stayer/you want to stay in the current kindergarten/you have staying intentions*).
B. I want to stay in the early care education field but would like to find a new job (*you are a mover/you want to move to a new kindergarten/you have moving intentions*).
C. I want to leave the early care education field and find a new job in a different field (*you are a leaver/you want to leave to a new field instead of an early care education field/you have leaving intentions*).

2: Occupational Commitment Scale

Based on the three-component conceptualization of Meyer and Allen (1991), this scale was compiled from three dimensions, namely, emotional commitment (liking the current occupation), normative commitment (being constrained by social norms), and continued commitment (leaving the current occupation will lose the corresponding benefits). This scale included 10 items, each rated on a Likert-type scale ranging from 1 (strongly disagree) to 5 (strongly agree). After the analysis, the Cronbach alpha coefficient of this scale concerning this study was 0.816, which showed good reliability and validity.

3. Psychological Resilience Scale

Psychological resilience scale in this study was measured in terms of both emotion regulation and coping strategies. Based on the Emotion Regulation Scale developed by Gross (1998), this scale was considered at two levels: cognitive reappraisal and expressive inhibition. Additionally, given the Brief COPE developed by Carver (1997), this scale was evaluated at three levels: planning, active coping, and instrumental support. After exploratory factor analysis, this part determined 14 items, and participants rated their consistency on a score of 1 (strongly disagree) to 5 (strongly agree) on a Likert-type scale. It showed good internal consistency with a Cronbach alpha coefficient of 0.804.

4. Job Satisfaction Scale

Currently, Hirschfeld (2000) dimensions of intrinsic (e.g., a chance to try out one’s ideas and feelings of accomplishment) and extrinsic (e.g., tangible aspects such as pay and working conditions) subscales are internationally recognized. Still, there is yet to be an agreement on classifying internal dimensions. To avoid indicator crossover with other determinants, through the discussion with experts, we designed the scale to include five dimensions, namely, job satisfaction (job match, occupational characteristics, autonomy); internal satisfaction (interpersonal relationships, working conditions, support system); reward satisfaction (remuneration and incentives, internal development); organizational management satisfaction (system construction, leadership behavior); and occupational environment satisfaction (policy support, social status, parental relationship). The scale included 24 items, and a Likert-type scale was used to rate the participants from 1 (strongly disagree) to 5 (strongly agree). The higher the score, the higher the job satisfaction. After the exploratory factor analysis, this scale deleted two items, and 22 remained. A revised Cronbach alpha coefficient was 0.862, indicating good internal consistency.

5. Maslach Burnout Inventory

The most widely used questionnaire for measuring burnout was MBI (Maslach et al., 1997), containing three different versions: MBI-Human Service Survey (MBI-SS), MBI-Educators Survey (MBI-ES) and MBI-General Survey (MBI-GS). The MBI-ES with good reliability and validity was used to investigate teacher burnout in this study, which is divided into three dimensions, namely emotional exhaustion (depletion of emotional resources, exhaustion, occupational stress), depersonalization (indifference in the relationship between the teacher and the client) and self-efficacy (the value of the individual to the job, competency). The MBI-ES consists of 14 items on a 5-point Likert-type scale ranging from 1 (strongly disagree) to 5 (strongly agree). The current study showed good internal consistency with a Cronbach alpha coefficient of 0.83.

### Data analysis

We conducted statistical analyses to examine the collected data comprehensively. We utilized SPSS 14.0 for descriptive statistics and correlations to offer an initial understanding of the dataset. In the preliminary analysis, we analyzed the data descriptively to summarize the relevant characteristics. Subsequently, we tested binary correlations between the main predictors and turnover intentions to explore the correlations between the variables. To clarify the risk rate of the predicted outcomes, we also conducted multinomial logistic regression analyses on the teachers’ turnover intentions (staying/moving/leaving). Meanwhile, we also utilized the logistic regression model and OLS regression of two turnover intention outcomes (staying or non-staying) to predict the direction and probability of teachers’ turnover intentions, assessing the predictive power of the regression models through standard errors. Finally, we also developed data models and trend plots based on the results of the above analyses.

## Results

### Basic information on teachers’ turnover intentions and predictors

There were 194 (approximately 21.1%) teachers intending to leave, 466 (about 21.1%) teachers intending to move, and the number of teachers who chose to stay was 466 (approximately 50.6%). Table 3 shows the mean levels of the four predictors and corresponding internal indicators. Psychological resilience had the highest mean value of 3.8. Occupational commitment was the next highest level, with a mean value of 3.68. The mean job satisfaction and burnout values were 3.61 and 3.06, respectively. The value of the four dimensions remained above the mean level.

**TABLE 3:** Correlations among predictors influencing teachers’ turnover intentions.

	1	2	3	4	5	6	7
1 Occupational commitment	1						
2 Psychological resilience	**0.**60**	1					
3 Job satisfaction	**0.**70**	**0.**66**	1				
4 Burnout	−0.33**	−0.242	−0.48**	1			
5 Turnover intention: leave	−0.44**	−0.18	−0.39**	0.41**	1		
6 Turnover intention: move	−0.34*	0.12*	−0.18	0.11	−0.32**	1	
7 Turnover intention: stay	0.38**	0.11	0.49**	−0.45**	−0.51**	−0.57**	1

*p < 0.05

**p < 0.01.

### Correlation analysis among predictors influencing kindergarten teachers’ turnover intentions

To investigate the correlation between the predictors and teachers’ turnover intentions, we further predicted the correlation coefficients among the predictors. Table 4 reflected the correlation coefficients among the predictors, indicating the linkage between the two variables to some extent. We could also observe the significance level according to the *P*-value. For example, the correlation coefficient between teachers’ occupational commitment and teachers’ turnover intention (leaving) was −0.438, which was significant at the 0.01 significance level. According to Table 4, there was a substantial correlation between predictors, which can be further analyzed.

**TABLE 4:** Regression analyses of predictors of teachers’ turnover intention.

	Move vs. stay	Leave vs. stay	Leave vs. move	Move/leave vs. stay	Turnover intention
Variables	RRR	RRR	RRR	OR	β
**Occupational Commitment**	0.51**	0.83**	0.13**	0.75**	−0.27**
Emotional regulation	2.53**	1.90*	0.12*	1.45*	0.09*
**Psychological Resilience**	2.45*	1.06*	0.02*	2.14*	0.25**
**Job satisfaction**	0.04	0.1**	0.36*	0.37*	−0.39**
Emotional exhaustion	1.09*	1.25*	0.71	1.13*	0.04*
Self-efficacy	0.11	2.67	1.75	1.68	0.07
**Burnout**	3.10**	1.02*	2.11**	1.35**	0.29**
Marital status	1.77	1.18	0.27	1.34	−0.09
Education level	0.71	0.76	0.94	0.70	0.02
Income status	1.41	0.82	1.25	0.92	−0.02
Type of position	1.66	1.93***	1.23	1.48	−0.07
Years of experience in the Kindergarten	1.48	1.23	1.62	1.00	−0.04
Years of experience in the Industry	0.99	1.21	0.49	1.38	−0.05
Type of kindergarten	0.40***	0.65	1.70	0.60***	0.17*
Establishment	4.65	1.04	0.88	1.03	−0.06

RRR, relative risk ratio (RRR); OR, odds ratio (OR); both values are based on the first group as the reference group.

*p < 0.05

**p < 0.01

***p < 0.001.

### Regression analysis of predictors of teachers’ turnover intentions

The first four columns in Table 4 compared turnover intentions: Move vs. Stay, Leave vs. Stay, Leave vs. Move, and Move/Leave vs. Stay. The first four columns describe the logistic regression analysis results, relative risk ratios describe the first three, and odds ratios represent the fourth. Finally, the fifth column in Table 4 describes the results of the OLS regression analysis, analyzing the correlation between predictors and teachers’ turnover intention after adjusting for covariates.

The first three columns in Table 4 also presented the logistic regression analysis results regarding relative risk ratio (RRR), and a relative risk ratio described each predictor variable. The fourth column predicted the odds ratio (OR) between Move/Leave and Stay. The relative risk ratio (RRR) indicates the change multiple of the event occurrence ratio when the observed predictor variable increases by one unit under the condition that other predictor variables remain unchanged. For example, the RRR of occupational commitment in Table 3 was lower than 1 (RRR = 0.51), which indicated that teachers would have a higher risk of the second outcome (Stay) compared to the first (Move). Specifically, a one-unit increase in teachers’ occupational commitment was associated with 0.51 times the risk of Move versus Stay, indicating that teachers are more likely to stay in their jobs rather than change jobs after adjusting for all other key predictors and covariates. Conversely, RRR greater than one meant that teachers were more likely to have the first outcome than the second. A relative risk ratio (RRR) equal to one means that teachers with these characteristics have a comparable risk or likelihood of having either outcome.

Table 4 showed that teachers with higher occupational commitment had a higher risk or likelihood of Stay over Move/Leave (RRR = 0.51 and 0.83 respectively, *p* < 0.01), and teachers were more likely to Move than Leave (RRR = 0.13). Teachers with greater psychological resilience predicted a higher risk of Moving and Leaving (RRR = 2.45 and 1.062, respectively, *p* < 0.05) and a higher likelihood of moving (RRR = 0.02) of the independent variables, greater emotional regulation predicted a higher risk of Moving and Leaving (RRR = 3.53 and 1.90 respectively, *p* < 0.05), and according to the third column, the risk of Moving was greater than that of Leaving. Additionally, teachers with higher burnout were more likely to have the outcome of Leave (RRR = 3.10 and 1.02), and the likelihood of Leave was twice as high as the likelihood of Moving. Regarding the internal structure, the stronger the teachers’ emotional exhaustion, the greater the risk of Moving or Leaving (RRR = 1.09 and 1.25, respectively, *p* < 0.05), and there was no statistically significant difference between the risk of Moving and Leaving for teachers. Moreover, teacher self-efficacy predicted no significant relationship or effect on teachers’ turnover intention. Finally, teachers with higher job satisfaction reported a lower risk of Leave (RRR = 0.04 and 0.1, respectively, *p* < 0.01).

Beta (β) in column five of Table 4 illustrated the association between the predictors and teachers’ turnover intentions, i.e., when β was positive, the predictors positively affected teachers’ turnover intentions. For example, teachers’ occupational commitment associated with teachers’ turnover intention (β = −0.27, *p* < 0.01) signified that greater occupational commitment reported an upward trend in turnover intentions. Conversely, psychological resilience associated with turnover intentions (β = 0.25, *p* < 0.01) indicated that psychological resilience had a significant positive effect on teachers’ turnover intention, i.e., the stronger the psychological resilience, the stronger the turnover intention. In addition, among the teachers’ demographic variables, including marital status, education level, income status, type of position, years of experience in the kindergarten, years of experience in the industry, type of kindergarten and establishment, only the type of kindergarten had a significant positive effect on teachers’ turnover intentions, that is, the statement that only teachers in private kindergartens had a higher likelihood of turnover was statistically valid.

## Discussion

### Move or leave

*Nearly 60% of kindergarten teachers prefer to Move rather than Leave when having turnover intentions*. In this research, nearly half (49.5%) of the kindergarten teachers surveyed had turnover intentions, consistent with existing studies. The proportion of kindergarten teachers intending to leave the field of early care education entirely accounted for 21%. Besides, 29% of kindergarten teachers had the intention to move and change jobs within the field of early care education, accounting for 59% of the teachers with turnover intentions. Based on the mentioned above, we could find that kindergarten teachers reported high turnover intentions, but 59% of them would choose to change to a new job in the same field of early care education rather than leaving this field entirely.

### Psychological resilience and turnover

#### The improvement of psychological resilience and working conditions could effectively mitigate the decision of kindergarten teachers to leave entirely

**FIGURE 1:**
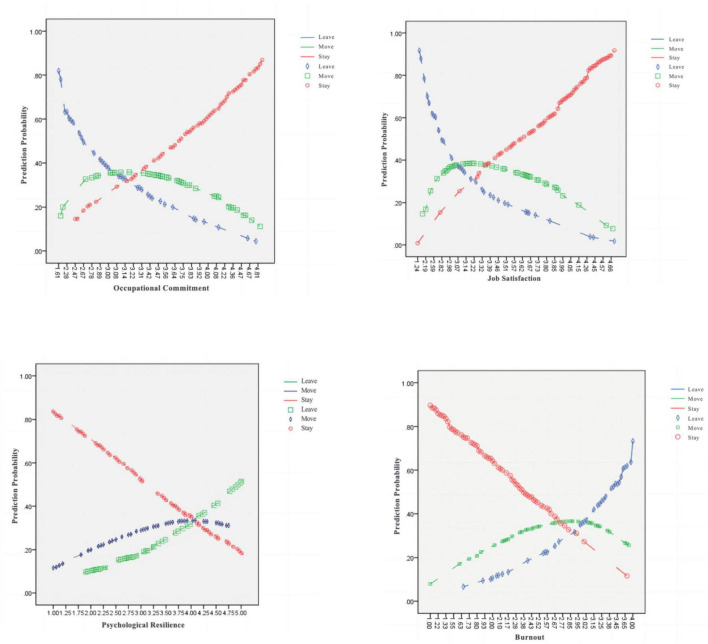
Predicted probability of turnover intention under each predictor.

What are the factors that influence more kindergarten teachers to move rather than to leave entirely? Through the in-depth analysis, we found the following factors to be the main reasons to mitigate the decision of kindergarten teachers to leave entirely. Firstly, teachers with greater psychological resilience are more likely to move, meaning that kindergarten teachers with better emotional regulation and coping strategies intend to change a new job within early care education rather than leave the field entirely (see Figure 1). Beltman et al. (2011) also suggested that psychological resilience may be the key to understanding why teachers leave the profession, given identical and often (dis)satisfying and stressful workplace conditions. Thus, hypothesis H2 is confirmed. As the prior study did not yet distinguish the turnover outcomes between move and leave, some findings were still limited to the result that psychological resilience negatively predicted turnover intentions (Hall-Kenyon et al., 2014). Evidently, this study effectively remedied the shortcomings of existing studies through improved research tools, and the process was rigorous.

Additionally, the possibility of such results has been reported in previous studies. For example, Chang (2013) noted that teachers with low coping skills tended to be more emotionally depleted and were more likely to seek emotional support and solutions actively (Wells, 2015). Thus, teachers with high planning skills, positive coping skills, and the ability to find tools to support themselves in the face of stress and depletion tended to change or improve their current job status by changing jobs. It was also suggested that providing teachers with the appropriate ways to deal with stress can help them stay in their original jobs.

On the other hand, kindergarten teachers with unsatisfactory working conditions were more likely to move within the field, especially under more stress caused by the working environment and context culture. Our findings were consistent with previous studies concerning turnover intentions and working conditions. Several researchers noted that good work conditions (e.g., clean and attractive) help teachers engage in their work more deeply (Arnup and Bowles, 2016; Chughtai and Zafar, 2006); however, teachers who work under adverse and unsatisfactory work conditions (e.g., noise and lack of teaching materials) have higher turnover intentions (Ladebo, 2005). This finding also suggested that reducing teachers’ occupational stress and strengthening school systems could mitigate the moving intention effectively. Relative studies by Hall-Kenyon et al. (2014), Wells (2015), and Whitebook et al. (2004) have shown that increased job stress and reduced job wellbeing would increase teachers’ turnover intentions. However, as these studies mentioned did not investigate moving separately from leaving, the results only indicated a simple trend, which was an improvement of this study. Moreover, this trend had been suggested by qualitative research, where teachers tended to be overwhelmed by sustained high levels of emotional exhaustion, and teachers feeling emotionally exhausted at work were more likely to seek a job in new fields rather than the field of early care education (Zhao, 2019). The reason for teachers’ turnover was not only caused by emotional exhaustion; its internal mechanism may be that teachers were dissatisfied with working conditions and produced excessive emotional exhaustion, but they lacked coping ability and were likely to change jobs (Totenhagen et al., 2016).

### Intrinsic motivation and turnover

#### Teachers with no turnover intentions are more concerned about intrinsic motivation, but there is a risk of “false retention

We found that intrinsic motivation could significantly affect teachers’ intention to stay in their current jobs compared to salary incentives, indirectly suggesting that teachers with greater intrinsic motivation are more likely to stay compared to extrinsic motivation. Relevant studies also showed that salary is not the primary factor when teachers have turnover intentions. Instead, the need for personal professional development was the critical factor affecting teachers’ intention to move within the field (Watt and Richardson, 2008). Additionally, while salary was an issue, low salary did not impact teacher turnover significantly (Zhao, 2019; Hanushek et al., 2004). The above findings suggested that we need to focus on improving the internal rewards system and pay attention to teachers’ intrinsic motivation and advancement. For example, opportunities for professional development and better administrative support (Bukhari et al., 2023), favored working conditions and environment (Abós et al., 2018), financial incentives and satisfactory salary (Harris, 2020).

Notably, concerning whether teachers with no turnover intentions were willing to stay in their current organization because of job satisfaction or occupational commitment, we found that suppression as an emotion regulation strategy was significantly related to teachers’ staying. It may imply that when teachers choose to stay in their current job, they will deal with the working problems and tasks through emotion suppression, difficulty dilution, or ignorance (Gross and John, 2003). Even if these teachers did not leave their current jobs, they were no longer mentally and emotionally part of the profession (Hughes, 2001). This was also a problem that often occurred in kindergartens. Teachers would spend their days chaotically and lifelessly, inevitably leading to carelessness for young children and a passive attitude in the face of their work, harming the construction of teacher teams.

### Job satisfaction and turnover

#### Job satisfaction has the strongest impact on teachers’ turnover intentions, but the performance is relatively poor

Among all factors affecting teachers’ turnover intentions, the explanatory strength of job satisfaction reached 42.7%. Existing studies revealed that higher job satisfaction predicted lower turnover intentions (Cooney, 2008; Granger and Marx, 1992; Seery and Corrigall, 2009). However, the mean value of this variable did not reach a relatively satisfactory level (3.61), especially the performance of reward satisfaction and practice environment satisfaction were the worst. According to Li and Wang (2018) study on teachers’ turnover intentions, life satisfaction was identified as the strongest influencing factor. Although its sample and comparison factors differed from the present research, it confirmed the important influence of job satisfaction on teachers’ turnover intentions. The performance of job satisfaction was unsatisfactory, which also suggested that improving job satisfaction was a top priority.

### Risk and protective factors and turnover

#### Risk and protective factors synergistically affect teachers’ turnover intentions

We found that occupational commitment and job satisfaction had an inverse effect on teachers’ turnover intentions, which fully proves the hypothesis H4. Teachers with occupational commitment reaching level three and above were more likely to stay in their current jobs (see Figure 1). In terms of internal indicators, teachers’ affective commitment, continuance commitment, and normative commitment all negatively predicted teachers’ turnover intentions, corroborating the studies of Wang et al. (2018) and Liu and Hai (2017) and supporting the hypothesis H1. This study also found that job satisfaction had an inverse effect on teachers’ turnover intentions, consistent with prior studies (Loncar, 2010). In the research concerning reasons for high teacher turnover rates in U.S. early care and education programs, Whitebook et al. (2004) found that teachers with negative evaluations of their working conditions and environments tended to leave for another childcare center or tended to seek new employment in a different career field where they could experience a more positive environment.

Moreover, we also found that all internal indicators reported a significant effect on teachers’ turnover intentions. In existing studies on indicators affecting turnover intentions, although the indicators were fragmented and scattered, they did provide support for the current research, e.g., salary and interpersonal relationships (Hall-Kenyon et al., 2014), internal development (Totenhagen et al., 2016), and staff stability (Whitebook et al., 2004) were positively related to teachers’ turnover intentions. In addition, we also found that teachers with higher autonomy and more support from the kindergarten were more significantly inclined to stay in their jobs, which was supported by the study of Torres (2014) concerning teachers’ increasing emphasis on autonomy and school voice.

Another finding of this study was that teachers with strong burnout and occupational psychology were more likely to leave the profession. Thus, hypothesis H3 is confirmed. This finding was consistent with Totenhagen et al.’s (2016) study. Totenhagen et al. (2016) explored the relationship between kindergarten teachers’ emotions and turnover intentions from a psychological perspective, showing that teachers’ negative emotions were negatively correlated with teachers’ turnover intentions. Finally, regarding internal indicators of burnout, teachers with higher levels of emotional exhaustion and occupational stress were more likely to leave the profession (see Figure 1).

## Conclusion

To sum up, this study investigated the correlation between occupational commitment, psychological resilience, job satisfaction, burnout, and turnover intentions of rural kindergarten teachers in China. The improvement of psychological resilience and working conditions could effectively mitigate the decision of kindergarten teachers to leave the field entirely. Teachers with no turnover intentions are more concerned about intrinsic motivation, but there is a risk of false retention. Job satisfaction has the strongest impact on teachers’ turnover intentions, but the performance is relatively poor. Risk and protective factors such as occupational commitment and job satisfaction synergistically affect teachers’ turnover intentions. These findings could provide inspiration and implications to mitigate kindergarten teachers’ turnover intentions.

## Limitations

By investigating kindergarten teachers in rural China, this study explored the relationship between occupational commitment, job satisfaction, psychological resilience, burnout, and turnover intentions. Some findings could provide theoretical and practical implications and suggestions for school and education departments. However, we should also note several limitations of the current study. First, the correlational methodology precludes definitive conclusions about directional relationships among variables. To address this, subsequent investigations could adopt experimental or longitudinal designs that can better delineate causal mechanisms. Second, the exclusive focus on educational contexts limits the generalizability of findings. Future scholarship could strengthen the model’s validity by investigating diverse sectors such as corporate training or vocational education environments.

## Data Availability

The raw data supporting the conclusions of this article will be made available by the authors, without undue reservation.
